# Inspiration, innovation and integration: highlights from the third ICPCN conference on children’s palliative care, 30 May to 2 June 2018, Durban, South Africa

**DOI:** 10.3332/ecancer.2018.870

**Published:** 2018-09-11

**Authors:** Julia Downing, Sue Boucher, Julia Ambler, Tracey Brand, Zodwa Sithole, Busi Nkosi, Michelle Meiring, Elizabeth Gwyther, Lorna Sithole, Barbara Steel, Alex Daniels

**Affiliations:** 1 International Children's Palliative Care Network (ICPCN), New Bond House, Bond Street, Bristol, BS2 9GA, UK; 2 Makerere University, Kampala, Uganda; 3 International Children's Palliative Care Network, Cluster Box 3050, Assagay, 3624, South Africa; 4 Umduduzi Hospice Care for Children, Durban, South Africa; 5 Hospice and Palliative Care Association of South Africa, Cape Town 7700, South Africa; 6 Palliative Treatment for Children (Patch) South Africa, Cape Town, 7700 South Africa

**Keywords:** palliative care, children, international, commitment, integration, education, research, South Africa, WHA resolution

## Abstract

The International Children’s Palliative Care Network (ICPCN) held its third international conference on children’s palliative care in Durban, South Africa, from May 30 2018 to 2 June 2018. The conference—inspiration, innovation and integration—brought together 250 participants from 41 countries and was held in conjunction with local partners—Umduduzi Hospice Care for Children, Palliative Treatment for Children South Africa (Patch SA) and the Hospice and Palliative Care Association of South Africa. It built on national and global developments in palliative care such as its inclusion in Universal health coverage (UHC), the Lancet Commission report on pain and palliative care and the sustainable development goals (SDGs), and aimed to raise the profile of children’s palliative care in KwaZulu-Natal (KZN) and nationally. Seven pre-conference workshops were held prior to the conference on topics such as pain and symptom management, children’s palliative care within a humanitarian crisis, perinatal palliative care, research, developing programmes, ethical issues and difficult conversations in children’s palliative care. Delegates were welcomed in true Durban style at the welcome reception hosted by the City of Durban and uShaka Marine World. The opening plenary included entertainment from the Open Air School and Hillcrest Primary School, and inspirational talks from the Member of the Executive Council (MEC) for Health, a representative of the World Health Organization (WHO), the Chief Executive of ICPCN and the Noble Peace Prize Nominee Dr MR Rajagopal from Pallium India. Plenary sessions were interspersed throughout the conference with 56 oral concurrent presentations and workshops, six ‘Meet the expert sessions’ 100 poster presentations and the South African Premier of the film ‘Hippocratic: 18 Experiments in gently shaking the world’. There was a great feeling of networking and learning throughout the conference, with the conference being well evaluated, and an increase in the level of presentations and research from previous conferences demonstrating the steps that are being taken in children’s palliative care globally.

## Introduction

The International Children’s Palliative Care Network (ICPCN) held its third international conference on children’s palliative care in Durban, South Africa. The conference was held at the Southern Sun Elangeni Hotel on Durban’s North Beach from 30 May 2018 to 2 June 2018, with pre-conference workshops on the 30 May 2018.

Much importance has been placed on the development of palliative care over the past few years since the signing of the World Health Assembly Resolution on Palliative Care in May 2014 [1], followed by the WHA Resolution on Cancer in May 2017 [2]. Alongside this, there is an ongoing emphasis on UHC [3] and the SDGs [4]. The WHO state that UHC means ‘that all people and communities can use the promotive, preventive, curative, rehabilitative and palliative health services they need, of sufficient quality to be effective, while also ensuring that the use of these services does not expose the user to financial hardship. It is firmly based on the WHO constitution of 1948 declaring health a fundamental human right and on the Health for All agenda set by the Alma Ta declaration in 1978. UHC cuts across all of the health-related SDGs and brings the hope of better health and protection for the world’s poorest’ [3]. This emphasis on UHC is important for the ongoing development and implementation of children’s palliative care, and was the theme for the 2017 World Hospice and Palliative Care Day—‘UHC and Palliative Care—Don’t leave those suffering behind’ [5] which also saw the launch of the Lancet Commission report ‘Alleviating the access abyss in palliative care and pain relief—an imperative of UHC: the Lancet Commission report’ [6]. With over 21.6 million children globally requiring palliative care [7], the Lancet Commission goes on to state that one-third of all children who died in 2015 experienced serious health-related suffering (SHS) with more than 98% of children needing palliative care and pain relief being from developing countries [6] such as those within sub-Saharan Africa. Thus, following on from the successes of their conferences in India and Argentina, ICPCN chose to hold their third international conference in Durban, South Africa, in order to raise the profile of children’s palliative care in the province of KZN and nationally.

The ICPCN aims to promote the development of children’s palliative care around the world. It is the only international organisation working as a global action network in the field of children’s palliative care and is acknowledged as the lead for children’s palliative care by the WHO and other international agencies [8–10]. The success of the ICPCN is built upon the sum of its parts as it is essential that members work together and collaborate in order to improve children’s access to palliative care throughout the world. At present, the network has 373 organisational members and 2,040 individual members from more than 125 countries. ICPCN’s vision is to live in a world where children’s palliative care is acknowledged and respected as a unique service and every child and young person with life-limiting or life-threatening conditions and their families can receive the best quality of life and care regardless of which country they live in. Their holistic needs encompass physical, emotional, spiritual and developmental aspects of care and ICPCN’s mission is, therefore, to attain the best achievable quality of life and care for children and young people with life-limiting conditions, their families and carers worldwide by raising awareness of children’s palliative care, advocating for the global development of children’s palliative care services and sharing expertise, skills and knowledge.

The organisation of an international conference is one way of implementing their mission, as participants from around the world can come and learn from each other. Thus, it was anticipated that this third International conference, held in South Africa, would promote this mission and also demonstrate the ongoing developments and evidence base for children’s palliative care, advocating for its development in sub-Saharan Africa and throughout the world.

## Palliative and cancer care for children in South Africa

South Africa is the southernmost country in Africa and the largest country in Southern Africa. In 2017, it had an estimated population of 56.52 million people, with 51% being women and 29.6 <15 years of age. KZN, where the conference was held, is the second largest province with a population of 11 million, the proportion of whom are <15 years is 21.1% [11]. In 2016, there were 320,000 children in South Africa living with human immunodeficiency virus (HIV), 55% who were receiving antiretroviral therapy with 12,000 new infections and 9,300 deaths [12]. The high burden of HIV disease alongside noncommunicable diseases such as cancer within sub-Saharan Africa, including South Africa, means that over 50% of the burden of SHS in children in low-income countries is associated with the HIV disease [6].

Whilst survival for children with cancer has increased globally to 75–80% in high-income countries, [13] this is not the case in low-middle income countries where survival rates are only 10–30% and account for 80% of childhood cancers [14–16]. The exact incidence of childhood cancer in sub-Saharan Africa is unknown due to the challenges in maintaining an accurate cancer registry. A recent population-based registry study on the international incidence of childhood cancer 2001–2010, covered just 0.8% of the population in sub-Saharan Africa due to the lack of the data available, however, South Africa contributed 70% of cases for the region reporting low incidence rates [17]. Childhood cancer is an emerging issue within the region. The first report from the South African Children’s Tumour Registry reported 11,712 childhood cancers in South African between 1987 and 2007 with an age-standardised average annual incidence rate was 45.2 per million, varying per ethnic groups ranging from 116 for whites to 37 for black African [18]. More recently, they identified the five most common childhood cancers in South Africa as : leukaemia, lymphoma, brain tumours, nephroblastomas or Wilms tumours and soft tissue sarcomas [19].

Whilst childhood mortality in South Africa has reduced over the past 30 years, a recent assessment of the need for palliative care for children in South Africa [7, 20, 21] estimated that between 600,000 and 800,000 children have palliative care needs, with more than 4,000 of these having cancer, with only 4.8% of those needing children’s palliative care accessing care in South Africa [20]. The HIV epidemic was the catalyst for the development of children’s palliative care in South Africa with the advent of Cotlands Hospice in Johannesburg in 1996. In 2007, a children’s palliative care portfolio was created within the Hospice and Palliative Care Association of South Africa (HPCA) which led onto the development of children’s palliative care training programmes funded by the Diana Princess of Wales Memorial Fund. Hospital-based children’s palliative care was started in 2006 through Bigshoes Foundation in Baragwanath Hospital, Gauteng, and later spread to two other provinces throughout the country. These programmes provided palliative care to children with all forms of life-limiting and life-threatening conditions. Through the University of Cape Town, South Africa has been running a Postgraduate Diploma/ Masters in Palliative Care since 2001, a children’s palliative care elective was introduced in 2010 with a separate children’s palliative care stream since 2017. In 2012, the National Children’s Palliative Care Network (Patch SA) was set up in order to mobilise and support a sustainable network of individuals, organisations, professionals and caregivers to provide holistic culturally appropriate palliative care for children and their families from diagnosis to bereavement. Since the WHA resolution on the palliative care the South Africa government has been committed to integrate palliative care into public healthcare, thus a palliative care policy was approved for implementation by the National Health Council in April 2017 (The National Strategic Framework for Palliative Care) [22]. The policy recognises the need to provide palliative care at the primary health care level through the re-engineering of the district health care programme and makes provision for the development of excellence at the tertiary level. Within the policy, children are recognised as needing special consideration and a task team is focusing exclusively on the needs of children, recognising that they have unique needs that need to be specifically planned and provided for [22].

## Pre-conference workshops

Seven pre-conference workshops were held on Wednesday 30 May. Over 200 participants attended the pre-conference workshops including doctors, nurses, social workers, volunteers, bereavement counsellors, religious leaders, students and teachers. Whilst many of the participants at the pre-conference workshop were from South Africa, with an emphasis on those from around Durban, there was a good international representation and the workshops provided an opportunity for sharing and learning from each other. Following on from the success of the workshop at the conference in Argentina, [23] a one-day workshop was held on managing pain and other symptoms in children’s palliative care from birth to young adulthood. Whilst pain is a common symptom, it, like other symptoms, is often under-treated, particularly in low- and middle-income countries, and emphasis needs to be put on closing this divide [6]. The workshop, therefore, focused on managing pain and symptoms in a variety of settings. Facilitated by paediatric pain experts from around the world (Dr Satbir Jassal UK, Dr Regina Okhuysen-Cawley Mexico/USA, Dr Michelle Meiring South Africa, Dr Catherine Habashy USA and Dr Pat Carragher UK), the workshop addressed issues of Comprehensive Symptom assessment, multidimensional pain management in children and managing other symptoms. The morning included presentations on different areas and was followed in the afternoon by four clinical stations—pain, dyspnoea, difficult conversations and integrative care. Finally, a case study of an adolescent patient with high symptom burden, existential distress, severe dyspnoea and delirium, along with disease progression was discussed in order to bring together the topics discussed during the day and apply them to practice. Links to various resources including the ICPCN learning programmes (www.elearnicpcn.org) [10], Basic Symptom Control in Paediatric Palliative Care [24], the 4th Edition of the APPM Master Formulary [25], the Really Practical Handbook of Children’s Palliative Care for Doctors and Nurses Anywhere in the World [26], Children’s Palliative Care in Africa [27] and the Oxford Textbook for children’s palliative care [28] were shared, and experiences discussed. Participants appreciated having experts from around the world facilitating the workshop, many of whom had contributed to the key resources shared.

With the enormous numbers of humanitarian crises globally, with over 128 million people requiring life-saving humanitarian assistance, [29] the importance of the provision of palliative care in humanitarian situations has been recognised [30]. To date, palliative care has often been missing in the response to such crises, but the recently re-written SPHERE guidelines have included palliative care as an integral part of the humanitarian response [31]. The morning workshop addressing children’s palliative care in humanitarian situations was well attended, with participants eager to share their experiences and discuss the role of palliative care and how best it can be provided. Having discussed the global need and the present palliative care response, both globally and in Africa, the situation of refugee children in Europe was explored, along with the palliative care needs assessment and response for Rohingyan refugees in Bangladesh. Finally, a case study was shared of an African refugee child and family and the challenges of providing children’s palliative care when families are divided due to a humanitarian crisis. The workshop was facilitated by Joan Marston (South Africa), Professor Danai Papadatou (Greece), Rachel Coghlan (Australia) and Lanice Shortell (USA).

Perinatal palliative care is a fast developing discipline, with the advent of frameworks, research and best practice programmes. The morning workshop led by facilitators from the UK (Dr Chakrapani Vasudevan) and Argentina (Dr Rut Kiman), discussed the need for, and models of palliative care in the perinatal and neonatal period [32], sharing examples of a perinatal pathway for babies with palliative care needs in the UK [33], creating a neonatal end-of-life palliative care protocol in the US [34] and the British Association of Perinatal Medicine framework for clinical practice in perinatal medicine [35]. Experiences of setting up perinatal/ neonatal services were discussed and opportunities given to discuss challenges and lessons learnt from around the world.

There is a dearth of research in children’s palliative care [36, 37], leading to the final morning workshop focussing on research in children’s palliative care. The workshop, facilitated by Professor Julia Downing (Uganda/UK), Dr Jan Aldridge (UK) and Marie Friedel (Belgium), explored issues regarding research in children’s palliative care including priorities for research, challenges to undertaking research in children’s palliative care, personal experiences of setting up a children’s palliative care research centre in the UK and undertaking a Ph.D. on children’s palliative care in Belgium. Examples of how research can be utilised at different levels of children’s palliative care development including at the global level, nationally through identifying a need, and locally at the service provision level were explored giving examples of ICPCN supported or led research. Recent calls to strengthen the evidence base for children’s palliative care [6, 36–38] were discussed, along with examples of where the lack of evidence in the field has been a challenge, for example in the development of the WHO guidelines on the pharmacological treatment of persisting pain in children with medical illnesses [39], where the lack of evidence in the field is cited as a challenge to guideline development [40]. Priorities for research into children’s palliative care were also discussed, utilising examples nationally, e.g. in Canada [41] and globally, e.g. the ICPCN Delphi study [42]. The experience of setting up a children’s palliative care research centre was shared along with that of undertaking a Ph.D. in children’s palliative care and opportunities given throughout for discussion and networking.

Three-afternoon workshops were also held. For many people at the conference, the issue of how to set up a children’s palliative care programme is important, with many facing challenges and uphill struggles. A workshop focussing on reaching out to develop a children’s palliative care programme, with facilitators from Butterfly House Children’s Hospice (Lynda Gould) in China and Hummingbird House in Australia (Paul Quilliam) addressed this. Key milestones were discussed, along with the need for community engagement, recognition of the need for children’s palliative care and the importance of integration of palliative care services within the national health system.

There are many challenging ethical situations in children’s palliative care, some of which have been highlighted in the media over the past 12 months, therefore to address these, a workshop on ethics in children’s palliative care facilitated by a team from the UK (Dr Richard Hain), South Africa (Dr Julia Ambler) and Romania (Dr Delia Birtar) was held. The interactive workshop provided the opportunity to examine the moral theories behind the four key ethical principles and their shortcomings, followed by an in-depth discussion around difficult ethical dilemmas and decision making commonly found in children’s palliative care. Individuals were divided into groups to work through different case studies highlighting opportunities for tension, differences in viewpoints, different moral perspectives and the human face of such dilemmas. Whilst the case studies gave real-life situations upon which to cite the discussion, it was clear that there were no easy answers, and that differences in opinion were common.

The final afternoon workshop was on difficult conversations in children’s palliative care—a topic relevant to everyone working within the field. Facilitated by a multi-disciplinary team from South Africa (Tracey Brand and Professor Hanneke Brits) and Australia (Dr Marianne Phillips) the workshop focused around breaking bad news, both to parents and to the children themselves. The challenges, tensions and stresses experienced in doing this were discussed and ways to overcome these shared. Participants were able to share their own experiences and how they cope with this, along with the importance of debriefing for professionals having broken bad news, and how we can support each other in this.

## Conference summary

**Figure 1.**
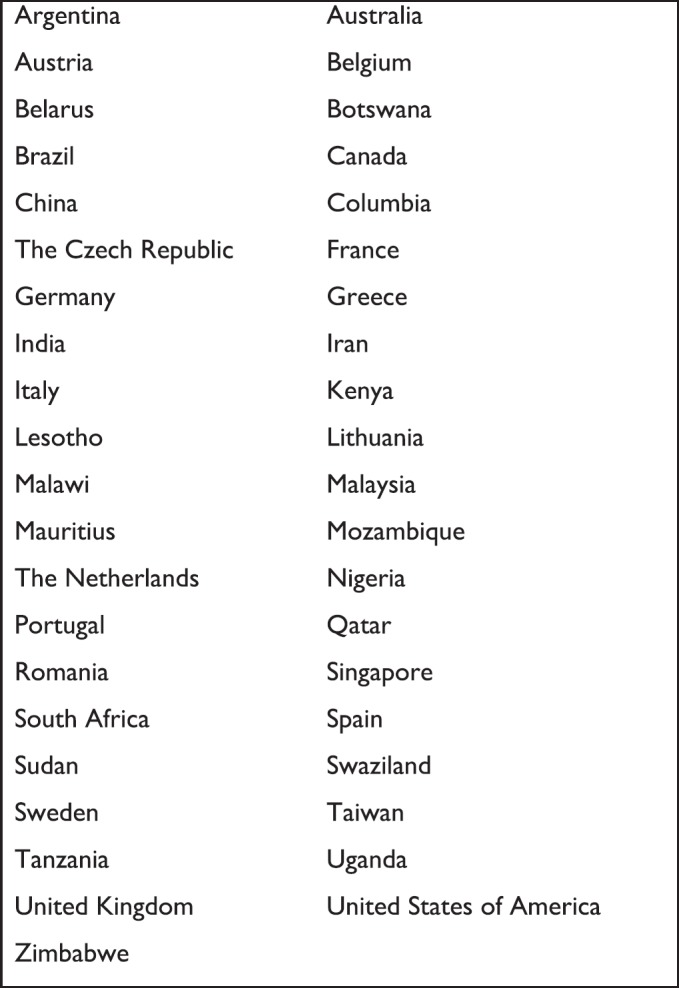
Countries from which delegates attended.

The conference, held at the Southern Sun Elangeni Hotel, brought together 250 participants representing 41 countries from around the world (Figure 1). The conference brought together a range of clinicians, advocates, academics, clergy, counsellors, dieticians, managers, pharmacists, researchers, social workers, teachers, policymakers, Ministry of Health officials, volunteers, play/art therapists and others working in the field of children’s palliative care.

The theme of the conference was inspiration, innovation and integration and gave participants the opportunity to be inspired by each other to celebrate innovation and explore the value of integration. The conference was run in collaboration with the local children’s palliative care programme, Umduduzi Hospice Care for Children, the National Children’s Palliative Care network—Patch SA and the national hospice and palliative care association—HPCA. The scientific programme included a variety of plenary sessions (15 papers and a film), 56 oral breakout presentations, including workshops, 100 poster presentations and six ‘meet the expert’ sessions. Oral and poster abstracts were accepted from 44 countries with representation from those new to the field and those who have been working in the field for many years, thus demonstrating the breadth of participants attending the conference. Workshops were held in areas such as: advancing paediatric medication access through policymaking; accompanying the suffering stranger; compassion in children’s palliative care; communicating for maximum impact around children’s palliative care; strategic planning for the African Children’s Palliative Care Network (ACPCN) and Pioneering Children’s Palliative Care. Meeting the expert sessions also covered areas such as managing complex situations, therapeutic touch, advocacy, psycho-social issues, building design in children’s palliative care and children’s palliative care in the South African Development Community countries. The conference also hosted the South African Film Premiere of the film *‘*Hippocratic: 18 Experiments in gently shaking the world’. The film, narrated by David Suchet, tells the story of Dr MR Rajagopal, and Indian doctor and 2018 Nobel Peace Prize Nominee, about his dream to provide universal access to essential and heavily restricted pain medicines in order to achieve a pain-free India.

Delegates were welcomed in true Durban style at the welcome reception on the evening of Wednesday 30th May. The welcome reception was hosted by the City of Durban at uShaka Marine World. Delegates were able to walk around the aquarium and view the different fish, including the sharks. Prior to the dignitaries, arriving delegates were given drinks and entertained to some South Africa singing. Delegates were then given a warm welcome to Durban and South Africa from a representative of the eThekweni Mayor, Cllr Zandile Gumede. This was followed by a welcome from the conference chair and ICPCN Chief Executive Professor Julia Downing, who then introduced Sister Frances Dominica, ICPCN Patron and the founder of Helen and Douglas House, who shared her experience of Inspiration, Innovation and Integration in developing children’s palliative care services. The value of hosting the conference in Durban and South Africa was recognised along with the South African Government’s commitment to palliative care through its recently approved policy. The reception was live and there was a real sense of networking and anticipation of the conference to come.

The conference got underway on Thursday morning to some singing and dancing from pupils from the Open Air School in Durban. The school caters for around 260 pupils from pre-primary to grade 12 and provides education to children with special education needs. They were followed by the drumming troupe from Hillcrest Primary School took to the stage—50 primary school pupils playing djembe drums of differing shapes and sizes. Both the choirs and drumming troupe established a powerful and emotional start to the conference, demonstrating the cultural mix and fusion found within Durban and KZN.

**Figure 2.**
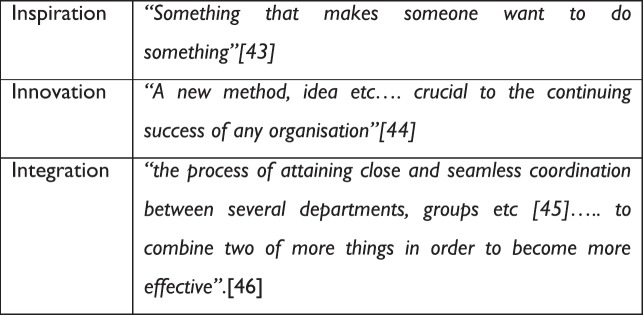
Definitions of inspiration, innovation and integration.

The MEC for Health, KZN, Dr Sibongeseni Dhlomo, and Chair of the National Steering Committee for the National Policy Framework and Strategy on Palliative Care opened the conference with his paper entitled ‘Palliative Care in South Africa: A commitment to Children’s Palliative Care’. He took participants through the development of children’s palliative care in South Africa, referring to Nelson Mandela’s statement ‘there can be no keener revelation of a society’s soul than the way in which it treats its children’ highlighting the importance that the South African government is putting on the development of children’s palliative care. Having shared developments, he closed by stressing the importance of the non-governmental organisation (NGO) sector working closely with the Government in order to work together to achieve more. It then fell to the ICPCN Chief Executive Professor Julia Downing to introduce the theme of the conference, and inspire delegates to think ‘outside of the box’, to be innovative and to see how they can integrate children’s palliative care into all that they do. Having introduced the global context, she broke down the theme into its three components—inspiration, innovation and integration (Figure 2). She challenged the participants to step out of their comfort zone during the conference and as they continue to develop children’s palliative care once they return home. Dr Marie-Charlotte Bouesseau from the WHO then joined the conference via Skype to talk about ‘Palliative Care for Children—it is time to make things happen’. She talked about the moral imperative/ethical duty of palliative care, the need to transform the modalities of care, moving from the ‘umbrella strategy’ [47] to building the ‘house’ of palliative care for the patients and their families, with the ‘house’ being an analogy to describe an integrated, people-centred approach and a cultural transformation of health services. She concluded by discussing what the WHO is doing with regard to children’s palliative care, including the publication of a guide for healthcare planners, implementers and managers on ‘Integrating Palliative Care and Symptom Relief into Paediatrics’, which is due for publication later in the year. Finally, Nobel Peace Prize Nominee Dr MR Rajagopal from Pallium India presented on integrating the results of the Lancet Commission into children’s palliative care [6]. He talked about the concept of SHS [6] and how the provision of palliative care and pain control will vary from place to place. He shared his experience of providing care in India, giving examples of where he provided pain control to those within the family, in order to reduce health-related suffering, and the inequalities that exist with regard to access to palliative care and pain control.

Following the opening session, participants attended concurrent sessions focusing on pain and symptom management, bereavement and nurses’ experience and a workshop on advancing paediatric medication access through policymaking. The workshop shared the experiences of South Africa in developing and implementing the policy, with papers presented at the other concurrent sessions from Nigeria, South Africa, Spain, Mexico, the United Kingdom, France, Australia, the Netherlands, Brazil and Qatar. It was exciting to hear from such a broad range of contexts, with a range of resources and experience. Whilst each setting is unique there were also similarities—for example, in issues such as pain control, supporting the bereaved and how nurses’ experience supporting parents and families in caring for dying children. The concurrent sessions after lunch continued to demonstrate innovations across the world. A session looking at outcomes and transitions in care was a great opportunity to share research in these areas, with lessons learnt from Africa being integrated into studies in Belgium and Spain, along with those from the UK. Similarly, the session on perinatal palliative care demonstrated the ways that palliative care is being integrated into perinatal/ neonatal care and the strides that have been made in the field since the last conference in 2016. Two workshops explored issues around compassion in children’s palliative care and how we accompany suffering families—whilst coming at the topic from different starting points the issue of compassions is central to all that we do within children’s palliative care.

Two other plenary sessions were held throughout the day—before lunch Kelly du Plessis, Founder of Rare Diseases South Africa, shared her experiences of being a mother of a child with Pompe disease, and the importance of integrating palliative care into the care of children with rare diseases. She talked about the journey that she had been on in terms of accessing palliative care, and when is the right time to do this, and how the fact that we are moving forward in the field of caring for children with rare diseases, this does not mean that we need to avoid introducing palliative care as ‘palliative care is a way of dealing with a situation that increases/betters the standards of life of patients jointly with their families, through stopping of (suffering) and the alleviation of pain and discomfort’. This fitted in with the earlier presentation by Dr Rajagopal about SHS. The day ended with the premier of the film ‘Hippocratic’, introduced by Mike Hill and Sue Collins of Moonshine Agency who produced the film. It was heart-warming to hear the story of Dr Rajagopal and his work to improve access to pain control and palliative care across India. Delegates were moved by Dr Rajagopal’s work and the way he has persevered over the years to bring comfort and relief of suffering to so many; in what have not been easy circumstances.

**Figure 3.**
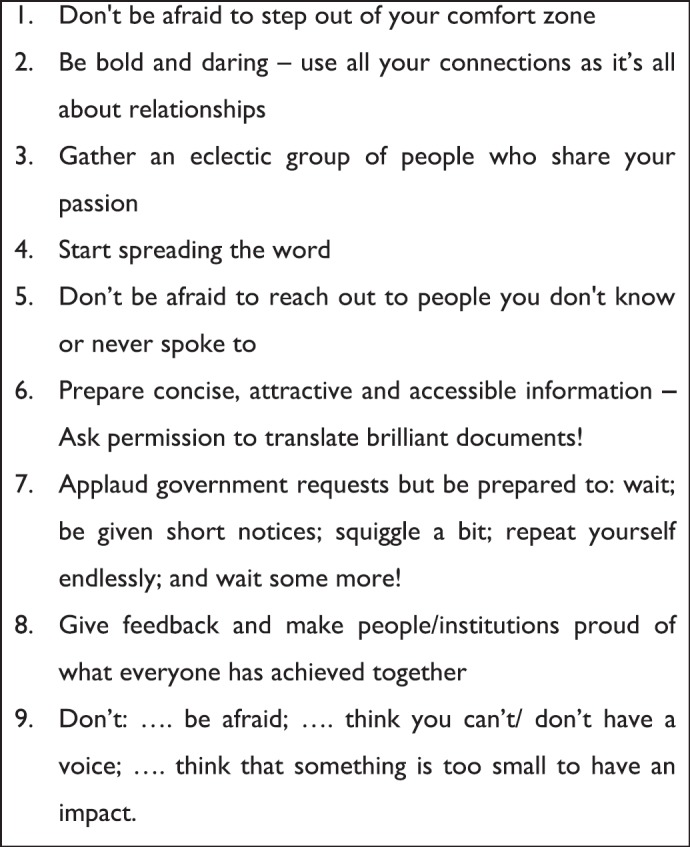
Lessons learnt in developing children’s palliative care in Portugal.

Following the meet the expert sessions, Friday morning’s plenary got off to a good start as delegates heard from direct stakeholders. First to speak was Huyaam Samuels, a young adult with a rare medical condition who has been the recipient of palliative care in South Africa for several years. She is passionate about children’s palliative care, believing it to be an essential and crucial part of living and giving children a chance to live life and not just exist. Following on from Huyaam, two groups of parents shared their experiences of losing children who were cared for through Umduduzi, a local children’s palliative care care provider. Their accounts were moving, as they courageously shared their feelings and grief, along with how they were supported by the palliative care team and the difference it made to them. Dr Michelle Meiring, Chair of Patch SA, continued the session by talking further about developing and introducing the palliative care policy in South Africa and the implications for the future development. Delegates were then inspired by Professor Ana Lacerda from Portugal who shared eight important lessons from her team’s experience of developing children’s palliative care in Portugal (Figure 3) —she followed on the theme from Professor Downing’s opening talk, urging delegates to step out of their comfort zone and see where that takes them. Dr Regina Ohkuysen-Cawley from the Latin American Palliative Care Association then shared their experiences of developing children’s palliative care in Latin America, citing examples from different countries within the region, building on the themes developed by previous speakers.

Concurrent sessions followed the inspirational morning plenary sessions covering topics such as children, young people and parents, service delivery and a workshop. The workshop focused around communicating for the maximum impact around children’s palliative care. Led by the ICPCN communications team and Sue Collins from Moonshine Agency, the workshop aimed to explore strategies for communication about children’s palliative care at the local, national, regional and international levels, looking at it from the perspectives of the media, the public, governments and health and social care professionals. The session looking at children, young people and parents was an opportunity to hear the voice of those directly involved in palliative care, through personal experience as well as research. Huyaam Samuels reported on a project looking at young people as direct stakeholder advocates and shared about the work with the Worldwide Hospice Palliative Care Alliance and the formation of palliative care voices which is a network of direct stakeholders—palliative care recipients and their carers contributing to global palliative care advocacy, and was set up in November 2017. The value and importance of the direct stakeholder voice have been recognised and it is hoped that this will be strengthened and develop further in the future. Within service delivery, delegates from Romania, South Africa, Australia and Hong Kong talked about different aspects of integrating palliative care into services such as within the prison system, extending from oncology to nononcology services, integrating it into the public health system, and the ambulance service, demonstrating the need to be innovative as we think about the integration of children’s palliative care.

The theme of service delivery was continued in the concurrent sessions after lunch, with experiences shared from South Africa, Rwanda, Kenya and Lesotho. It was exciting to see how children’s palliative care has been integrated into different settings and how children’s palliative care is being embraced by a wide variety of organisations, such as within the government system in Rwanda, Kenya and Lesotho, and through NGOs (alongside the government) across South Africa. The importance of communication in children’s palliative care was stressed within another of the concurrent sessions, looking at issues of communication from Jordan, the UK, Tanzania, Brazil and South Africa. Innovative ways of communication were explored, including the use of social media such as WhatsApp (South Africa) and cyberspace in Brazil. The use of play as a therapeutic tool was also discussed and a review of the literature presented. Alongside these two concurrent sessions, there was a strategic planning meeting for the ACPCN—this network is aimed at strengthening advocacy for children’s palliative care within Africa and is jointly chaired by the ICPCN and the African Palliative Care Association.

Three other plenary presentations were given throughout the day. In the first, Dr Pat Carragher, Medical Director of Children’s Hospices Across Scotland who discussed innovative models of transitioning from children’s to adult palliative care services. He shared the model used in Scotland and drew out some lessons that could be useful for those looking at the issue of transitions, so that they could learn from the mistakes of others. The theme continued as Dr Satbir Jassal, General Practitioner and Medical Director of Rainbows Hospice for Children and Young Adults, looked at some of the innovations in the pharmacological treatment of pain. He explored issues around the principles of pain management, distress versus pain, some of the specific medications used, routes of drug administration and finally the evidence base. He stressed the need for the ongoing development of an evidence base for pain management in children, whilst sharing existing research and guidance. Finally, Professor Danai Papadatou, Professor of Clinical Psychology at the National and Kapodistrian University of Athens, and President of the NGO Merimna, shared her experience and the literature on accompanying the dying child and family to the end-of-life. Her presentation was inspirational, as she looked at what it means to ‘accompany’ someone, to be their ‘companion’. We need the ability to ‘hold and contain suffering’ and to facilitate exploration of unfamiliar situations through empowering, guiding and encouraging [48]. She finished by talking about the ‘rippling’ effect and how this refers to ‘the fact that each of us creates—often without our conscious intent or knowledge—concentric circles of influence that may affect others for years, ever for generations’ (Irvin Yalom) [49].

The final morning started well with three meet the expert sessions which were followed by a workshop on pioneering children’s palliative care, and two concurrent sessions on networks and history and service delivery. The workshop was an opportunity for delegates to discuss some of the essential ingredients in pioneering new children’s palliative care services. Facilitators from China, Australia, Romania, USA and South Africa shared their experiences prior to the discussion around the issues involved. The theme of service delivery continued on from the previous day looking at integrating children’s palliative care in schools (Uganda), midwifery (Argentina), home-based care (Malawi), an academic tertiary hospital (Singapore) and nationally (Zimbabwe). Alongside this, a session was held looking at the role and importance of networks such as the ICPCN, the European Association of Palliative Care children’s palliative care taskforce, the ACPCN and the Childhood Cancer Network in KZN, South Africa. The importance of strengthening these networks was discussed along with the influential role that they can have at the national and policy levels. Some ongoing work on the global history of children’s palliative care was also shared, demonstrating the multi-faceted and multi-national influences on the global children’s palliative care agenda.

The conference drew to a close with a final plenary session which looked at the status of children’s palliative care in KZN. Dr Neil McKerrow, the Head of Paediatrics and Child Health for KZN reviewed the demographics of children in the province, the health services and then how children’s palliative care has been integrated into the existing health system in order to strengthen the provision of children’s palliative care in KZN. Finally, Linda Ganca, Social Worker and Lecturer at the University of Cape Town, readdressed the theme of inspiration, innovation and integration through the role of education in children’s palliative care. She issued a challenge by stating that children’s palliative care education has the capacity to do much more than just train professionals to look after children with life-limiting conditions. Suggesting that if properly done by professionals who integrated lived experiences, children’s palliative care education can achieve social justice in health and be a catalyst to heal the many wounded healers and carers working in the field.

As the conference drew to a close, Sabine Kraft, the Chair of the ICPCN, launched the children’s life walk. This is a challenge for a worldwide pilgrimage throughout every participating country, from ICPCN member to ICPCN member, accompanied by the ‘Angel torch’, in order to bring attention to all children suffering from a life-limiting illness. The children’s life walk was launched in Germany by the National Association of Children’s Hospices in Germany—the ‘Kinder-Lebens-Lauf’. As she summed up the conference, Professor Julia Downing brought the conference to a close by thanking all those involved, presenting prizes to the two top posters, and reminding participants to be innovative and inspirational and demonstrated the integration of children’s palliative care into all that they do—she reminded us that ‘if you want something in your life you’ve never had you’ll have to do something, you’ve never done’ (JD Houston).

Throughout the conference, there was a sense of excitement, networking and moving forward. Whilst there is still an ongoing need for the development of evidence within the field [36] it was clear that much progress has been made over the past couple of years and that the quality of the papers—both oral and poster was higher, with more attention to research and evaluation, from a wider range of countries. Prior to the conference, and indeed during the conference, there was much evidence of social media activity through both Facebook and Twitter, but also of the national and international press. Various articles were published in the local newspapers with regard to children’s palliative care, radio interviews were held—both pre-recorded and live, and the Chief Executive of ICPCN along with the Noble Peace Prize Nominee Dr MR Rajagopal, were interviewed live on TV by the South African Broadcasting Company. The final day of the conference was International Children’s Day, which linked in well to children’s palliative care and provided a platform for us to speak.

**Figure 4.**
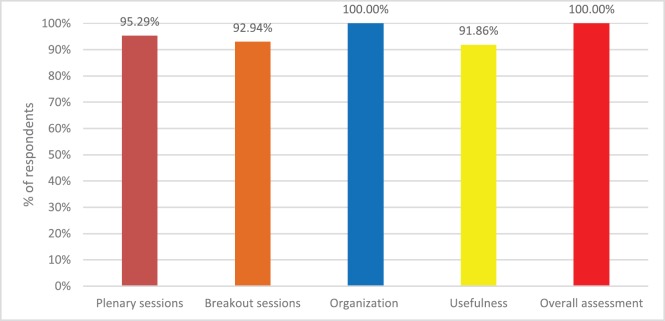
Percentage respondents scoring good and very good for each area.

A post-conference evaluation was completed by 86 delegates (35%) from 25 countries, including 31 doctors (36%) and 24 nurses (28%). Respondents were asked to rate plenary sessions, breakout sessions, organization of the conference, usefulness to work situation and overall assessment of the conference on a scale of 1 to 5, with 1 = very bad and 5 = very good. The results were encouraging with Figure 4 showing the percentage of respondents scoring 4 or 5 in each area. A wide variety of comments were made about the conference including that it was a ‘very well organized conference with a suitable number of participants that made networking very efficient’, that there was an ‘inspiring attitude throughout’ and that individuals felt part of something bigger than just their own country and that they were not alone. One participant notes that ‘most presentations were very inspiring and useful’ and that there was a good combination of ‘plenary sessions to inspire and concurrent sessions to inform’. Suggestions for topics for future conferences were made including debating some key issues. Encouragingly one participant wrote: ‘I thought the level of presentations and research much improved on previous years, the research was more relevant and useful to practice, a very well balanced programme and range of topics, really well done’.

## Conclusion

It was great to see the progress that has taken place with regard to the development of children’s palliative care globally. It was also good to see that the standard of the research presented, abstracts and papers was much higher than on previous occasions. This third international conference run by the ICPCN served as a unique opportunity to share with others working in the field and to explore issues of innovation, inspiration and integration. The conference venue was abuzz with chatter and activity during the tea and lunch breaks with delegates mixing, viewing posters and enjoying the local cuisine. The feelings engendered by the conference were captured by one delegate who wrote ‘Never before have I felt so much at home at a conference. Never before have I been surrounded by so many brilliant, kind, compassionate, unassuming and warm souls all at once. People who refuse to take no for an answer, for whom no wall is too high, no boundary impervious, no childless important than the others. People who insist on looking for the humanity that unites us all’.

## Conflicts of interest

The authors declare that they have no conflicts of interest.

## Funding

No funding was received for the preparation and publication of this report.

## References

[1] World Health Assembly (2014). Strengthening of palliative care as a component of integrated treatment within the continuum of care.

[2] World Health Assembly (2017). Cancer prevention and control in the context of an integrated approach.

[3] WHO (2018). Definition of Universal Health Coverage. http://www.who.int/health_financing/universal_coverage_definition/en/.

[4] UNDP sustainable development goals (2015). UNDP. https://www.undp.org/content/dam/undp/library/corporate/brochure/SDGs_Booklet_Web_En.pdf.

[5] WHPCA Universal health coverage and palliative Care—Don’t leave those suffering behind.

[6] Knaul FM, Farmer PE, Krakauer EL (2017). Alleviating the access abyss in palliative care and pain relief—an imperative of universal health coverage: the Lancet Commission report. Lancet.

[7] Connor SR, Downing J, Marston J (2017). Estimating the global need for palliative care for children: a cross-sectional analysis. J Pain Symptom Manage.

[8] Marston J, Boucher S, Downing J (2013). International Children’s Palliative Care Network: working together to stop children suffering. Eur J Palliat Care.

[9] Downing J, Boucher S, Nkosi B (2014). Transforming children’s palliative care through the International Children’s Palliative Care Network. Int J Palliat Nurs.

[10] Marston J, Boucher S, Downing J (2018). International Children’s Palliative Care Network: a global action network for children with life-limiting conditions. J Pain Symptom Manage.

[11] Stats SA (2017). Mid-year population estimates 2017.

[12] UNIADS Country factsheets: South Africa 2016. http://www.unaids.org/en/regionscountries/countries/southafrica.

[13] McGregor LM, Metzger ML, Sanders R (2007). Pediatric cancers in the new millennium: dramatic progress, new challenges. Oncology.

[14] Pritcahrd-Jones K, Sullivan R (2013). Children with cancer: driving the global agenda. Lancet Oncology.

[15] Magrath I, Steliarova-Doucher E (2013). Pediatric cancer in low income and middle-income countries. Lancet Oncol.

[16] Eden T (2018). Childhood cancers in low and middle-income countries: prevention and potentially curable treatment?. Cancer Control.

[17] Steliarova-Foucher E, Colombet M, Ries LAG (2017). International incidence of childhood cancer, 2001–10: a population-based registry study. Lancet Oncol.

[18] Stefan DC, Stones DK, Wainwright RD (2015). Childhood cancer incidence in South Africa, 1987–2007. S Afr Med.

[19] CANSA (2018). http://www.cansa.org.za/types-of-childhood-cancer/.

[20] Connor SR, Sisimayi C (2013). Assessment of the need for palliative care for children: three country report South Africa, Kenya and Zimbabwe.

[21] Connor SR, Sisimayi C, Downing J (2014). Assessment of the need for palliative care for children in South Africa. Int J Palliat Nurs.

[22] National Health Council (2017). Policy Framework and Strategy on Palliative Care 2017–2022 (Draft).

[23] Downing J, Kiman R, Boucher S (2016). Children’s palliative care ….. Now! highlights from the second ICPCN conference on children’s palliative care. Ecancermedicalscience.

[24] Jassal SS (2016). Basic symptom control in paediatric palliative care. Together for Short Lives and the Rainbows Hospice for Children and Young Adults.

[25] Jassal SS (2017). The Association of Paediatric Palliative Medicine Mater Formulary.

[26] Amery J (2016). A Really Practical Handbook for Children’s Palliative Care for Doctors and Nurses Anywhere in the World.

[27] Amery J (2009). Children’s Palliative Care in Africa.

[28] Goldman A, Hain R, Liben S (2012). Oxford Textbook of Palliative Care for Children.

[29] United Nations Office for the Coordination of Humanitarian Affairs (OCHA) (2017). Global Humanitarian Overview 2017: a consolidated appeal to support people affected by disaster and conflict.

[30] Powell RA, Schwartz L, Nouvet E (2017). Palliative care in humanitarian crises: always something to offer. Lancet.

[31] The Sphere Project (2017). Handbook for Humanitarian Response Draft 2.

[32] Balaguer A, Martin-Ancel A, Ortigoza-Escobar D (2012). The model of palliative care in the perinatal setting: a review of the literature. BMC Pediatrics.

[33] Dickson G (2017). A Perinatal Pathway for Babies with Palliative Care Needs.

[34] Catlin A, Carter B (2002). State of the art: creation of a neonatal end-of-life palliative care protocol. J Perinatol.

[35] BAPM (2010). Palliative Care a Framework for Clinical Practice in Perinatal medicine. Report of the Working Group.

[36] Downing J (2016). Editorial: to research or not to research—an important question in paediatric palliative care. Palliat Med.

[37] Beecham E, Hudson BF, Oostendorp L (2016). A call for increased paediatric palliative care research: identifying barriers. Palliat Med.

[38] Harding R, Sheer L, Albertyn R (2010). The Status of Paediatric Palliative Care in Sub-Saharan Africa—An Appraisal.

[39] World Health Organisation (2012). WHO guidelines on the pharmacological treatment of persisting pain in children with medical illnesses.

[40] Milani B, Magrini N, Gray A (2011). WHO calls for targeted research on the pharmacological treatment of persisting pain in children with medical illnesses. Evid-Based Child Health.

[41] Baker JN, Levine DR, Hinds PS (2015). Research Priorities in pediatric palliative care. J Pediatr.

[42] Downing J, Knapp C, Mukaden MA (2015). Priorities for global research into children’s palliative care: results of an International Delphi Study. BMC Palliative Care.

[43] (2018). Definition of Inspiration.

[44] (2018). Definition of Innovation.

[45] (2018). Definition of Integration.

[46] (2018). Definition of Integration.

[47] Stjernsward J, Foley KM, Ferris FD (2007). The public health strategy for palliative care. J Pain Symptom Manage.

[48] Papadatou D (2009). In the face of death: professionals who care for the dying and the bereaved.

[49] Yalom I (2008). Staring at the Sun: Overcoming the Dread of Death.

